# Optimizing *Escherichia coli* strains and fermentation processes for enhanced L-lysine production: a review

**DOI:** 10.3389/fmicb.2024.1485624

**Published:** 2024-10-04

**Authors:** Zijuan Wu, Tianpeng Chen, Wenjun Sun, Yong Chen, Hanjie Ying

**Affiliations:** ^1^National Engineering Research Center for Biotechnology, College of Biotechnology and Pharmaceutical Engineering, Nanjing Tech University, Nanjing, China; ^2^State Key Laboratory of Materials-Oriented Chemical Engineering, College of Biotechnology and Pharmaceutical Engineering, Nanjing Tech University, Nanjing, China; ^3^Soochow University, Suzhou, China

**Keywords:** L-lysine, *Escherichia coli*, genome modification, fermentation optimization, separation and purification

## Abstract

lysine is an essential amino acid with significant importance, widely used in the food, feed, and pharmaceutical industries. To meet the increasing demand, microbial fermentation has emerged as an effective and sustainable method for L-lysine production. *Escherichia coli* has become one of the primary microorganisms for industrial L-lysine production due to its rapid growth, ease of genetic manipulation, and high production efficiency. This paper reviews the recent advances in *E. coli* strain engineering and fermentation process optimization for L-lysine production. Additionally, it discusses potential technological breakthroughs and challenges in *E. coli*-based L-lysine production, offering directions for future research to support industrial-scale production.

## 1 Introduction

L-lysine is an essential amino acid that plays a crucial role in the growth and development of both animals and humans, particularly in protein synthesis, bone development, immune function, and hormone production (Matthews, 2020). Since animals cannot synthesize lysine on their own and must obtain it through diet or supplements, L-lysine is especially important as a feed additive in the livestock industry (Bampidis et al., 2022). Additionally, lysine is widely used in the food processing industry and the pharmaceutical sector, such as in the production of health products, medicines, and nutritional supplements (Cheng et al., 2018; Pandey et al., 2020). In 2021, the global lysine market was valued at $6.96 billion, and it is projected to reach $12.38 billion by 2030, with a compound annual growth rate of 6.5% (Leinonen et al., 2019). The continuous growth in global demand for L-lysine is mainly driven by the increasing consumption of meat and the rising demand for high-protein foods (Wang L. et al., 2021). According to market research, the growth of the lysine market is primarily concentrated in the Asia-Pacific, North America, and Europe regions, with particularly strong demand in areas with advanced livestock industries (Parisi et al., 2020). In recent years, lysine production has gradually shifted from traditional chemical synthesis methods to biological fermentation methods to meet market demands for environmental protection and sustainable development (He et al., 2024; Félix et al., 2019).

In industrial production, L-lysine is primarily produced through microbial fermentation (Ikeda, 2017). Microbial fermentation for L-lysine production offers multiple advantages: first, this method utilizes microbial metabolic pathways to efficiently synthesize L-lysine through the fermentation process, resulting in high production efficiency; second, the fermentation method is relatively environmentally friendly, reducing potential pollutants generated during chemical synthesis; third, microbial fermentation can use renewable resources as raw materials, thereby lowering production costs; and finally, through genetic engineering techniques, microbial strains can be further optimized to enhance the yield and purity of L-lysine (Anaya-Reza and Lopez-Arenas, 2017; D'Este et al., 2018). These advantages make microbial fermentation the preferred method for L-lysine production, bringing significant economic benefits while meeting modern industrial requirements for environmental protection and sustainable development.

The main strains used in microbial fermentation for L-lysine production include *Escherichia coli*, *Corynebacterium glutamicum*, and *Brevibacterium flavum*. Among these, *Escherichia coli* has become the dominant strain for L-lysine production due to its well-characterized genetic background, extensive gene editing tools, and short growth cycle (Mir et al., 2024; Chen et al., 2013). The different methods for producing L-lysine in *Escherichia coli* are shown in Table 1.

**Table 1:** Different methods for L-lysine production in *Escherichia coli*.

Modulation	Feature	Titer (g/L)	Time (h)	Culture Style	References
Methyl nitronitrosoguanidine mutagenesis	High-yield mutant strain screening	15	48	Batch	Nadeem et al. (2001)
Knockout of *rmf* gene	Inhibit protein synthesis to reduce stress on cell growth	4.465	48	Batch	Imaizumi et al. (2005)
Increase glyceraldehyde-3-phosphate dehydrogenase activity	More energy and intermediates	16.9	72	Batch	Yuri and Yuri (2010)
Multiple *dapA* gene mutations	Alleviate feedback inhibition of L-lysine	9	48	Fed-batch	Geng et al. (2013)
Optimization of cultivation conditions (DO, osmotic stress, glucose, ammonium sulfate, acetate)	Optimize culture conditions	134.9	72	Fed-batch	Ying et al. (2014)
Knockout of *ldcC* and *succ*, overexpression of *dapA*, aspartokinase evolution	Prevent L-lysine conversion to putrescine	0.67	48	Fed-batch	Wang et al. (2015)
High-throughput screening method	High-yield mutant strain screening	136.51	48	Fed-batch	Wang et al. (2016)
GREACE-assisted ALE	High-yield mutant strain screening	155	42	Fed-batch	Wang et al. (2019)
Overexpression of meso-diaminopimelate dehydrogenase	Redirect diaminopimelate pathway	119.5	40	Fed-batch	Xu et al. (2019)
Enzyme-constrained model, NH^4+^ and O_2_ regulation	Optimize protein expression and metabolism	193.6	–	Fed-batch	Ye et al. (2020)
*gadE, hdeB, sodB, katE* with different promoter strengths	Acid tolerance module	69.3	48	Fed-batch	Yao et al. (2022)
Screening marker with rare codons	High-yield mutant strain screening	14.8	48	Fed-batch	Liu et al. (2023)
Multiple mutagenesis and cell fusion	High-yield mutant strain screening	53.8	24	Fed-batch	Li et al. (2023)
Knockout of *mlc* and heterologous *malAP* expression	Improved disaccharide and trisaccharide utilization	160	36	Fed-batch	Xu et al. (2024)

In recent years, significant progress has been made in the mutagenesis breeding of L-lysine-producing *E. coli* (Tadepally, 2019). Through physical, chemical, and biological mutagenesis methods, researchers have been able to induce genetic mutations in *E. coli* and screen for mutants with high lysine production. UV irradiation and chemical mutagens such as nitrosoguanidine (NTG) are commonly used in mutagenesis breeding (Wang J. et al., 2021; Xu Z. et al., 2016). High-throughput screening can rapidly identify mutant strains with high L-lysine production (Zeng et al., 2020; Kaczmarek and Prather, 2021). With the development of metabolomics, transcriptomics, and other technologies, the efficiency of mutagenesis breeding has continuously improved, allowing researchers to more accurately analyze and screen for mutants with desirable traits. However, due to the randomness of mutagenesis, mutagenesis breeding is often combined with genetic engineering to further optimize strain performance (Chen et al., 2023).

Genetic engineering breeding plays an increasingly important role in the improvement of L-lysine-producing *E. coli* (Xu et al., 2015). Through gene editing, metabolic engineering, and synthetic biology techniques, researchers can precisely modify the metabolic pathways of *E. coli* to enhance its lysine production capacity (Liu et al., 2022). Common strategies include the overexpression of key enzyme genes, the knockout of competing metabolic pathways, and the optimization of transcriptional regulatory networks (Dasgupta et al., 2020). By utilizing gene editing technologies such as CRISPR-Cas9, scientists can simultaneously modify multiple genes to achieve global optimization of metabolic networks (Wu et al., 2020). Genetic engineering breeding has already been applied in industrial production, significantly improving lysine production efficiency. In the future, by integrating intelligent methods such as machine learning and metabolic modeling, genetic engineering breeding is expected to further enhance the efficiency and automation of lysine production (Xu et al., 2020).

Optimizing the fermentation process of *E. coli* for lysine production is a crucial step in improving production efficiency. Current research focuses primarily on the composition of the culture medium, fermentation parameters, and process control (Du Y. H. et al., 2022). By optimizing the carbon-to-nitrogen ratio, trace elements, and vitamins in the culture medium, the growth rate of *E. coli* and lysine production can be significantly increased (Hao et al., 2020). Additionally, controlling temperature, pH, and dissolved oxygen levels during fermentation can effectively maintain the metabolic activity of *E. coli* and reduce the generation of by-products (Wang et al., 2018). Furthermore, the application of novel fermentation processes, such as continuous fermentation and fed-batch fermentation, has achieved positive results in industrial production, allowing for extended fermentation times and increased lysine accumulation. In recent years, intelligent fermentation systems have gradually emerged, utilizing sensors and automated control technologies to achieve real-time monitoring and dynamic adjustment of the fermentation process, further enhancing production efficiency and process stability (Egbune et al., 2024; Chen T. et al., 2019). These advancements have laid a significant foundation for the large-scale industrial production of lysine in *Escherichia coli*.

This paper reviews the research progress on enhancing L-lysine production in *Escherichia coli* through strain engineering and fermentation optimization. It also highlights newly developed strategies for L-lysine production in *E. coli*, as well as various value-added chemicals derived from L-lysine. The review provides future directions for improving L-lysine yields through innovations in systems biology and bioprocess engineering.

## 2 Strain modification

### 2.1 Traditional mutation breeding

Mutagenesis breeding of L-lysine-producing *Escherichia coli* began in the mid-20th century when scientists started using physical and chemical mutagens, such as ultraviolet (UV) radiation, X-rays, and N-methyl-N′-nitro-N-nitrosoguanidine (NTG), to induce genetic mutations in *E. coli* to screen for strains with enhanced L-lysine production (Nakamori, 2017). Early applications of this method successfully developed several industrially valuable mutants, significantly improving L-lysine production capacity. For instance, by mutagenizing *E. coli* with NTG, researchers were able to screen for L-lysine auxotrophic strains (Lys-), followed by penicillin enrichment to eliminate wild-type strains (Choi et al., 2016). Additionally, UV and ethyl methanesulfonate (EMS) mutagenesis, both individually and in combination, were employed to screen for L-lysine auxotrophic mutants, optimizing mutagenesis conditions (Liu and Jiang, 2015). As technology advanced, the efficiency of mutagenesis breeding continued to improve, especially with the integration of modern high-throughput screening techniques, allowing researchers to more rapidly identify superior mutants. This has enabled the continued application of this traditional breeding method in L-lysine production (Wang et al., 2012).

Currently, research on the mutagenesis breeding of L-lysine-producing *E. coli* is ongoing, with a focus on enhancing mutagenesis efficiency, screening for superior high-yield mutants, and further optimizing the metabolic pathways of mutants through metabolic flux analysis (Hirasawa and Shimizu, 2016). Although mutagenesis breeding involves randomness and unpredictability, its simplicity, ease of implementation, and low cost make it an important tool in industrial microbial breeding (Li et al., 2022; Sanghavi et al., 2020). In the future, with the advancement of genome editing technologies and systems biology, mutagenesis breeding is expected to combine with precise genetic engineering methods, such as CRISPR-Cas9, to achieve more efficient targeted mutagenesis and further increase L-lysine production. Additionally, the GREACE method (Genomic Replication Engineering Assisted Continuous Evolution) is an evolutionary engineering approach used to enhance microbial tolerance and production capabilities (Lee and Kim, 2020). The core principle of this method involves the introduction of proofreading-deficient mutants of the DNA polymerase complex, which promotes continuous mutations *in vivo*, thereby accelerating the evolutionary process. In practical applications, the GREACE method employs a “mutation combined with selection” strategy to rapidly screen for strains with superior phenotypes under stress conditions (Zhao et al., 2022; Luan et al., 2013). For instance, in studies aimed at improving L-lysine production, an *E. coli* strain RS3 modified using the GREACE method achieved a final yield of 155.0 g/L, with a glucose conversion rate of 0.59 g/g and a total output of 605.6 g in a 5-liter fermenter (Wang et al., 2019). This breeding strategy, which integrates traditional and modern technologies, will provide new directions for the industrial application of *E. coli* in L-lysine production (Nakamori, 2017).

### 2.2 Genetically engineered breeding

Genetic engineering of strains is a comprehensive process involving the knockout of specific genes, targeted mutagenesis, overexpression of key enzyme genes, and the modification of metabolic pathways. Through precise modifications and optimizations, the lysine production capacity of *Escherichia coli* can be significantly enhanced, providing highly efficient and stable strain resources for industrial production (Leong et al., 2020).

#### 2.2.1 Biosynthesis pathway of lysine in *Escherichia coli*

**Figure 1:**
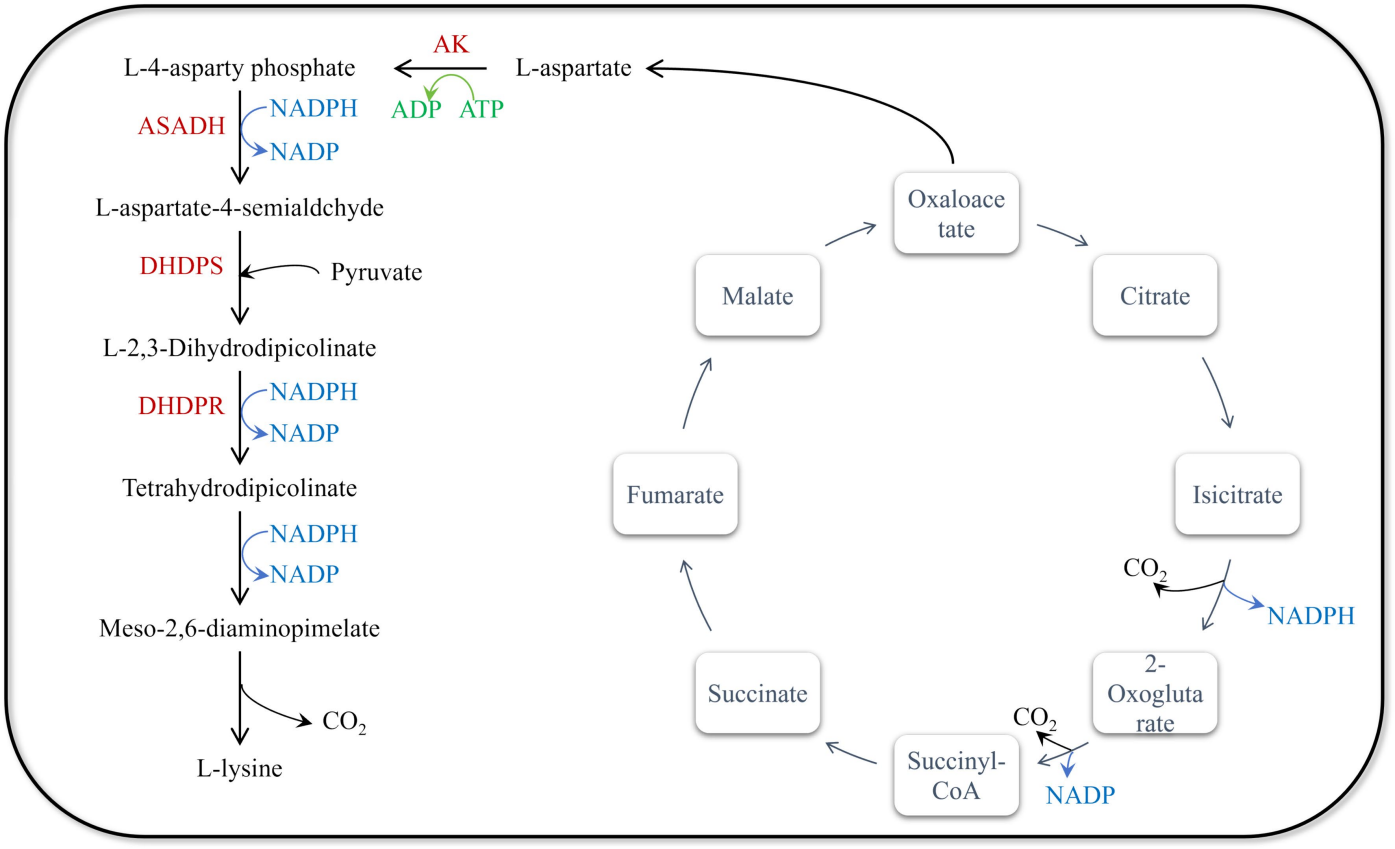
Synthesis pathway of L-lysine in *Escherichia coli*. There are six enzymes related to L-lysine biosynthesis in *E. coli*: ASADH (aspartic semialdehyde dehydrogenase, encoded by asd), AK (aspartic kinase, encoded by lysC), DHDPR (dihydropyridine dicarboxylic acid reductase, encoded by dapB), DHDPS (dihydropyridine dicarboxylic acid synthase, encoded by dapA), DAPDC (diaminopimelate decarboxylase, encoded by lysA) and DAPDH (diaminopimelate dehydrogenase, encoded by ddh).

The genomic modification strategies for lysine production in *Escherichia coli* are primarily designed based on the lysine biosynthesis pathway in *E. coli* (Figure 1). The synthesis of lysine begins with L-aspartate, which is catalyzed by aspartate aminotransferase (AST) from oxaloacetate. Oxaloacetate is a key intermediate in the tricarboxylic acid (TCA) cycle. Under the catalysis of aspartate kinase (AK), L-aspartate first reacts with ATP to form L-4-asparty phosphate, a crucial initial step in the aspartate pathway. Subsequently, L-4-asparty phosphate undergoes a series of enzymatic reactions to convert into L-aspartate-4-semialdehyde, which is then catalyzed by dihydrodipicolinate synthase (DHDPS) to produce L-2,3-dihydrodipicolinate, a key intermediate in lysine biosynthesis. L-2,3-dihydrodipicolinate is then reduced by dihydrodipicolinate reductase (DHDPR) to tetrahydrodipicolinate. Through a series of enzymatic reactions, tetrahydrodipicolinate is ultimately converted into meso-2,6-diaminopimelate (DAP). This process involves several enzymes, including DAP aminotransferase and DAP decarboxylase. Finally, DAP decarboxylase catalyzes the conversion of meso-2,6-diaminopimelate into L-lysine.

#### 2.2.2 Gene knockout

Gene knockout breeding is a method of increasing the lysine production of *Escherichia coli* through genetic engineering (Theisen and Liao, 2017). This process typically involves targeted knockout or mutation of certain genes in the *Escherichia coli* genome to optimize metabolic pathways, reduce byproduct production, and increase lysine production. By knocking out metabolic pathway genes that compete with lysine, such as tyrosine and tryptophan synthesis genes, as well as key enzyme genes involved in lysine degradation pathways, the production of by-products can be reduced, lysine consumption can be reduced, and more carbon can be directed toward lysine synthesis and accumulation can be increased (Ohno et al., 2014). By knocking out or mutating feedback inhibition genes (such as *dapA*) and enhancing key enzymes in the lysine synthesis pathway (such as *LysA*), the efficiency of lysine synthesis can be further improved. By reducing the generation of by-products such as acetic acid and lactic acid, optimizing the metabolic network, and introducing new metabolic pathways, lysine production can be further optimized (Liu et al., 2023).

Gene editing techniques, including CRISPR/Cas9 and *λ*—Red homologous recombination, have been applied to modify the genome of *Escherichia coli* (Zhao et al., 2016). Composite gene editing methods have also been explored, such as combining lambda Red recombination with homologous endonucleases I-SceI or CRISPR/Cas9. Although CRISPR-Cas9 is commonly used for gene knockout, altering multiple genes remains challenging (Su et al., 2020; Feng et al., 2018). Researchers combined CRISPR-Cas9 and MetClo assembly to design a standardized iterative genome editing system for *Escherichia coli*. This method simplifies the process of introducing multiple gene modifications, making it more efficient (Dong et al., 2021). These techniques have enabled researchers to study gene function and optimize metabolic pathways to produce high-value products.

#### 2.2.3 Targeted mutagenesis and promoter replacement

Targeted mutagenesis refers to the precise mutation of specific genes in the *Escherichia coli* genome using gene-editing technologies to enhance lysine biosynthesis or reduce lysine degradation pathways (Simon et al., 2018). By mutating key enzyme genes in the pathway, it is possible to decrease by-product formation or increase the activity of critical enzymes. For example, mutating the gene encoding phosphoenolpyruvate carboxylase (PPC) can reduce pyruvate consumption, thereby increasing the supply of lysine precursors (Shimizu and Matsuoka, 2022; Yin et al., 2024). Alternatively, mutating genes encoding regulatory factors can alleviate feedback inhibition in lysine biosynthesis. For instance, in the L-lysine biosynthesis pathway, targeted mutagenesis of the *lysC* gene, which encodes aspartokinase, and the *dapA* gene, which encodes dihydrodipicolinate synthase, can relieve feedback inhibition by L-lysine on its biosynthetic pathway (Li et al., 2023).

Promoter replacement is a method used to regulate the expression of target genes by replacing the promoters of genes in *Escherichia coli* (Marschall et al., 2017). By replacing the original weak promoter with a strong promoter, the transcription level of the target gene can be increased, thereby enhancing lysine synthesis (Jin et al., 2019). For example, replacing the promoter of the *dapA* gene with the constitutive tac promoter can achieve stable and efficient gene expression (Ying et al., 2014). Through promoter engineering, promoters that are sensitive to environmental conditions (such as temperature, pH, and nutrients) can be designed, enabling dynamic regulation of lysine synthesis (Wang et al., 2020).

#### 2.2.4 Overexpression of key enzyme genes

Overexpressing key enzyme genes in the lysine biosynthesis pathway can enhance the activity of these enzymes, thereby increasing lysine production (Li et al., 2017). Aspartokinase is a crucial regulatory enzyme in this pathway, whose activity is subject to feedback inhibition by lysine (Lo et al., 2009). By constructing a feedback-resistant mutant of *lysC* and overexpressing this gene in *Escherichia coli*, this feedback inhibition can be alleviated, leading to increased lysine production. Dihydrodipicolinate synthase is the rate-limiting enzyme in lysine biosynthesis, and its overexpression can significantly accelerate lysine synthesis. In addition to *lysC* and *dapA*, overexpression of genes such as *dapB*, *dapD*, and *lysA* can also enhance the efficiency of lysine synthesis (Zhang et al., 2023). For instance, Xu J. et al. (2016) studied key enzymes in the L-lysine ester synthesis pathway of a high-yield L-lysine strain, including dihydrodipicolinate synthase (DHDPS) and aspartokinase III (AK III). They modified these enzymes to protect them from feedback inhibition and enhance their activity.

#### 2.2.5 Optimization of metabolic pathways

**Figure 2:**
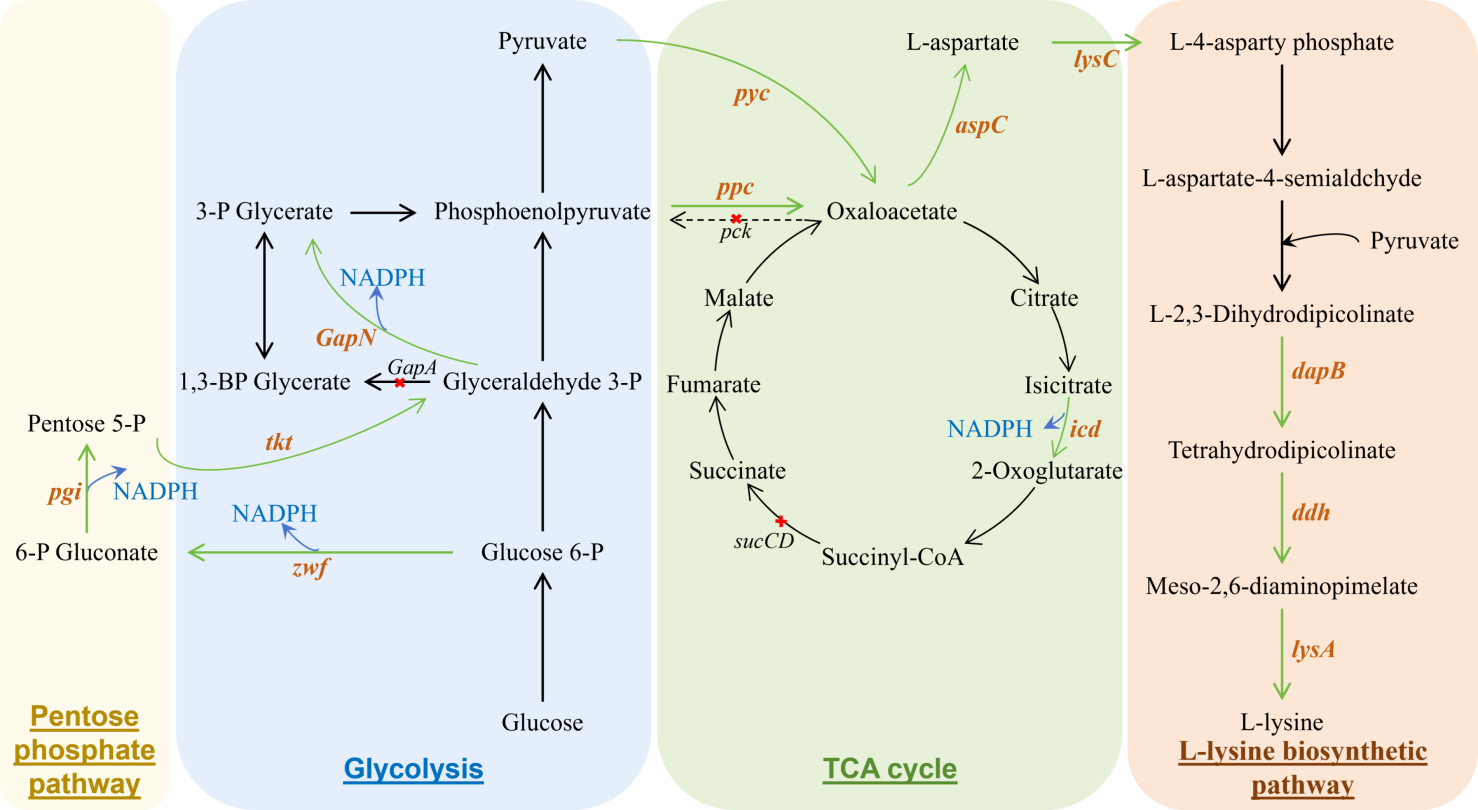
Metabolic engineering modification of *Escherichia coli* to produce L-lysine. The green arrows indicate the enhancement of the synthetic pathway by overexpressing the relevant enzyme gene; the dashed line with “X” indicates the inactivated response. These genes encode the following enzymes: *pgi*, glucose-6-phosphate isomerase; *zwf*, Glucose-6-phosphate 1-dehydrogenase; *tkt*, Transketolase; *pck*, Phosphoenolpyruvate carboxykinase; *pyc*, Pyruvate carboxylase; *ppc*, Phosphoenolpyruvate caroxylase; *icd*, Isocitrate dehydrogenase; *sucCD*, succinyl-CoA synthetase; *aspC*, Aspartate aminotransferase; *ddh*, Diaminopimelate dehydroganase; *lysA*, Diaminopimelate decarboxylase.

In *Escherichia coli*, lysine metabolism involves the tricarboxylic acid (TCA) cycle, glycolytic pathway, diaminopimelate pathway, and pentose phosphate pathway (Figure 2). By modulating the carbon flux ratio between the glycolytic and pentose phosphate pathways, the production of NADPH can be increased, which favors lysine biosynthesis (Tsuge and Kondo, 2017; Wendisch, 2017). Additionally, the introduction of a heterologous multifunctional enzyme, which replaces multiple original steps of enzyme-catalyzed reactions, can simplify the lysine biosynthetic pathway, thereby enhancing production efficiency. Furthermore, increasing the concentration of lysine synthesis precursors within the metabolic pathway can effectively boost lysine yield (Banno et al., 2018).

#### 2.2.6 Coenzyme balance

In the lysine biosynthesis pathway, NADH and NADPH are crucial cofactors. Optimizing the balance of these cofactors can significantly enhance lysine production (Wang et al., 2017). By introducing or overexpressing NADH oxidase, the accumulation of NADH can be reduced, thereby increasing the availability of NADPH. Researchers can also optimize the NADPH/NADH ratio by modulating the cellular cofactor regeneration systems (Liu et al., 2018). Alternatively, by knocking out or downregulating metabolic pathways that competitively consume NADPH, more NADPH can be directed toward lysine biosynthesis. For example, knocking out certain genes involved in fatty acid synthesis in *Escherichia coli* can reduce NADPH consumption. Lysine synthesis requires a substantial amount of ATP. Enhancing ATP generation pathways can also improve lysine production. For instance, overexpressing ATP synthase can increase intracellular ATP levels (Gao et al., 2021). Moreover, through metabolic flux analysis (MFA), researchers can quantitatively analyze the intracellular flow of cofactors and strategically adjust metabolic pathways to optimize lysine production.

In summary, genome editing technologies hold great promise for boosting lysine production in *E. coli*, benefiting industries such as animal feed and biotechnology. Researchers continue to refine these methods to improve strain performance and yield (Chen et al., 2024a; Eggeling et al., 2015).

### 2.3 Omics analysis and computer simulation

Omics analysis and computational simulation techniques have played a pivotal role in the breeding research of L-lysine-producing *Escherichia coli* (St. John and Bomble, 2019). These technologies provide a novel perspective for understanding and optimizing the metabolic pathways of *E. coli*. Through multi-layered Omics analyses, including genomics, transcriptomics, proteomics, and metabolomics, researchers can comprehensively reveal the key genes, regulatory networks, and metabolic bottlenecks that influence L-lysine production in *E. coli*. This information is crucial for designing more efficient L-lysine-producing strains (Becker and Wittmann, 2018). For instance, metabolomics analysis allows researchers to identify significant intermediate metabolites in the L-lysine production process and their accumulation patterns, thereby enabling gene modifications to enhance production efficiency (Ahmed et al., 2024).

On the other hand, computational simulation techniques, particularly metabolic network modeling and dynamic simulations, have further enhanced the precision of breeding efforts. Using these tools, researchers can predict the potential impacts of metabolic engineering strategies, assess the effectiveness of different genetic modifications, and conduct virtual experiments to reduce blind spots and time costs in actual experiments (Wang L. et al., 2021). In recent years, machine learning-based computational simulation tools have also begun to be applied in optimizing L-lysine-producing strains. These tools analyze vast amounts of Omics data to predict the optimal gene modification targets (Costello and Martin, 2018).

Manipulating single or multiple key genes within a metabolic pathway to increase the production of desired metabolites requires several rounds of experimentation to identify enzymes that impact productivity or yield (Chen et al., 2018). The utility of mathematical tools can effectively accelerate this process. Ensemble modeling (EM) utilizes phenotypic data (such as the impact of enzyme overexpression or knockout on steady-state yield) to select plausible models that can describe the available data, thereby improving L-lysine-producing strains. Recently, a strategy was explored for forming a set of dynamic models that capture the cellular dynamics through phenotypic data screening, followed by consecutive enzyme overexpression, which demonstrated some predictive capacity. Referring to the resulting strain (overexpression of desensitized diaminopimelate synthase *dapA*), this set of models could predict the desensitization of aspartokinase *lysC* as a subsequent rate-controlling step in the L-lysine pathway. This work illustrates the usability, applicability, and broad application of an ensemble modeling framework for constructing production strains (Brockman and Prather, 2015). Recently, researchers have also developed an innovative computational strategy that combines flux balance models with statistical methods, proposing a method to redirect flux by analyzing correlations in flux within the metabolic network to optimize product yield (Lee and Kim, 2015).

### 2.4 Innovative technology

#### 2.4.1 Biosensor

The application of biosensors in breeding lysine-producing *Escherichia coli* offers a novel, rapid, and sensitive method for screening and optimizing high-efficiency strains. Biosensors typically consist of a receptor element that specifically binds to the target product, such as lysine, and a reporter element that generates a measurable signal (Newman et al., 2011). By integrating these sensors into the genome of *E. coli*, researchers can monitor lysine concentrations in real time and select high-producing strains based on signal intensity. This approach significantly reduces the cumbersome quantitative detection steps in traditional high-throughput screening processes and greatly improves breeding efficiency. For example, Wang et al. (2016) utilized an L-lysine sensor in *E. coli* to screen mutant strain MU-1 from an atmospheric and room temperature plasma (ARTP) mutagenesis library, achieving a 9.05% increase in conversion rate and a 21% increase in yield compared to the original strain.

In recent years, significant progress has been made in the field of biosensor breeding for lysine-producing *E. coli*, driven by advancements in synthetic biology and gene editing technologies. Recent research has focused on developing more precise and multifunctional biosensors. For instance, one type of sensor is based on the transcriptional regulator *LysG*, which responds to high concentrations of L-lysine by promoting the expression of the *lysE* gene (Steffen et al., 2016). By fusing the *lysE* gene with a fluorescent protein gene and using fluorescence-activated cell sorting (FACS) to select cells with strong fluorescence signals, high L-lysine-producing strains can be obtained (Ye et al., 2020).

Another type of sensor is based on the interaction between L-lysine and the L-lysine aptamer riboswitch, which results in a regulatory effect (Shi et al., 2018; Wang et al., 2016). For instance, Yang et al. (2013) used an L-lysine riboswitch to regulate the expression of *tetA*. When the intracellular concentration of L-lysine is high, the expression of *tetA* is blocked, rendering the cells sensitive to tetracycline. *TetA* can transport Ni^2+^ into the cytoplasm, leading to cell death. If its expression is blocked, Ni^2+^ intracellular transport is prevented, allowing the cells to grow in a NiCl_2_ environment. By selecting an appropriate concentration of NiCl_2_ as a selection pressure, strains with high L-lysine production can be screened. They used this biosensor to optimize the promoter of phosphoenolpyruvate carboxylase (encoded by the *ppc* gene), which plays a key role in the distribution of metabolic flux in L-lysine production, and identified promoters from a constructed *ppc* promoter library that favored L-lysine ester production (Wang W. et al., 2022).

Moreover, researchers have developed multiplex biosensor systems capable of detecting multiple metabolites or intermediate products simultaneously, providing a more comprehensive assessment of metabolic pathway optimization (Chu et al., 2022). Another important advancement is the integration of machine learning with biosensors, allowing for the analysis of large datasets to predict and design more efficient sensor systems. The combination of these technologies has greatly enhanced the potential applications of biosensors in the breeding of lysine-producing strains, further advancing the production efficiency of lysine in *E. coli*.

#### 2.4.2 High-throughput screening

In recent years, the combination of lysine biosensors and fluorescence-activated cell sorting (FACS) has established a classical high-throughput screening strategy (Della Corte et al., 2020). This system enables the screening of cells containing plasmid libraries as well as cells with genomic mutations. For instance, the study by Wang Yan et al. introduced a novel approach for evolving industrial lysine-producing strains using an L-lysine-inducible promoter. Based on intracellular enhanced green fluorescent protein (EGFP) levels controlled by the promoter, fluorescence-activated cell sorting was initially used to screen 186 strains from a library of 10 million mutants derived from a high-lysine-producing *Escherichia coli* strain. A multiparameter evaluation of lysine concentration, yield, and cell-specific productivity in these strains identified two mutants, MU-1 and MU-2, which achieved lysine yields of 136.51 ± 1.55 g/L and 133.29 ± 1.42 g/L, respectively, in a 5-liter fermenter. Compared to the original strain, the lysine concentration and yield of the MU-1 strain increased by 21.00 and 9.05%, respectively, while those of the MU-2 strain increased by 18.14 and 10.41%, respectively. The mutant selection and evaluation system developed in this study can be applied to continuously enhance lysine strains for industrial production (Fang et al., 2024; Schendzielorz et al., 2014).

Additionally, droplet microfluidics technology allows for high-throughput screening in tiny droplets. Using Mass-Activated Droplet Sorting (MADS), cell variants with high lysine production can be screened based on signal intensity measured by electrospray ionization mass spectrometry (ESI-MS) (Payne et al., 2023). Strategies based on tRNA modification can be used to effectively identify and screen strains with high amino acid yields (Blay et al., 2020). For example, a research group at Beijing Institute of Technology proposed a strategy called AMINO, which screens high-producing amino acid strains based on synthetic tRNA CUA. This strategy utilizes aminoacylation reactions to reduce the affinity of tRNA for aminoacyl-tRNA synthetase by modifying the anticodon of tRNA, thereby screening for strains with high yields of the target amino acid (Guo et al., 2023). Other studies have employed large-scale activated droplet sorting (MADS) technology to screen *E. coli* mutants growing in microdroplets in a high-throughput manner. This method selects droplets based on signal intensity measured by electrospray ionization mass spectrometry (ESI-MS), enabling screening at a rate of 0.5 samples per second, thus rapidly identifying high-lysine-producing mutants (Payne et al., 2023).

#### 2.4.3 Directed evolution

Directed evolution simulates the natural selection pressures *in vitro*, allowing *Escherichia coli* to gradually adapt to high-lysine production environments, thereby selecting mutant strains capable of maintaining high growth and production levels under elevated lysine concentrations (Matsui and Asano, 2015). Recently, Xurong Yao and colleagues designed a synthetic acid-tolerance module to finely regulate the expression of multifactorial gene blocks. Through directed evolution, they developed an acid-responsive *asr* promoter library, from which four variants were selected for constructing the synthetic module. The laboratory strain *Escherichia coli MG1655* was first selected for growth under mildly acidic conditions (pH 5–6), followed by an evaluation of the lysine production performance of the industrial lysine-producing *E. coli* strain *SCEcL3*. The results demonstrated that this synthetic biology strategy significantly enhanced the robustness and productivity of both strains in mildly acidic environments (Yao et al., 2022).

#### 2.4.4 Microbial symbiont design

**Figure 3:**
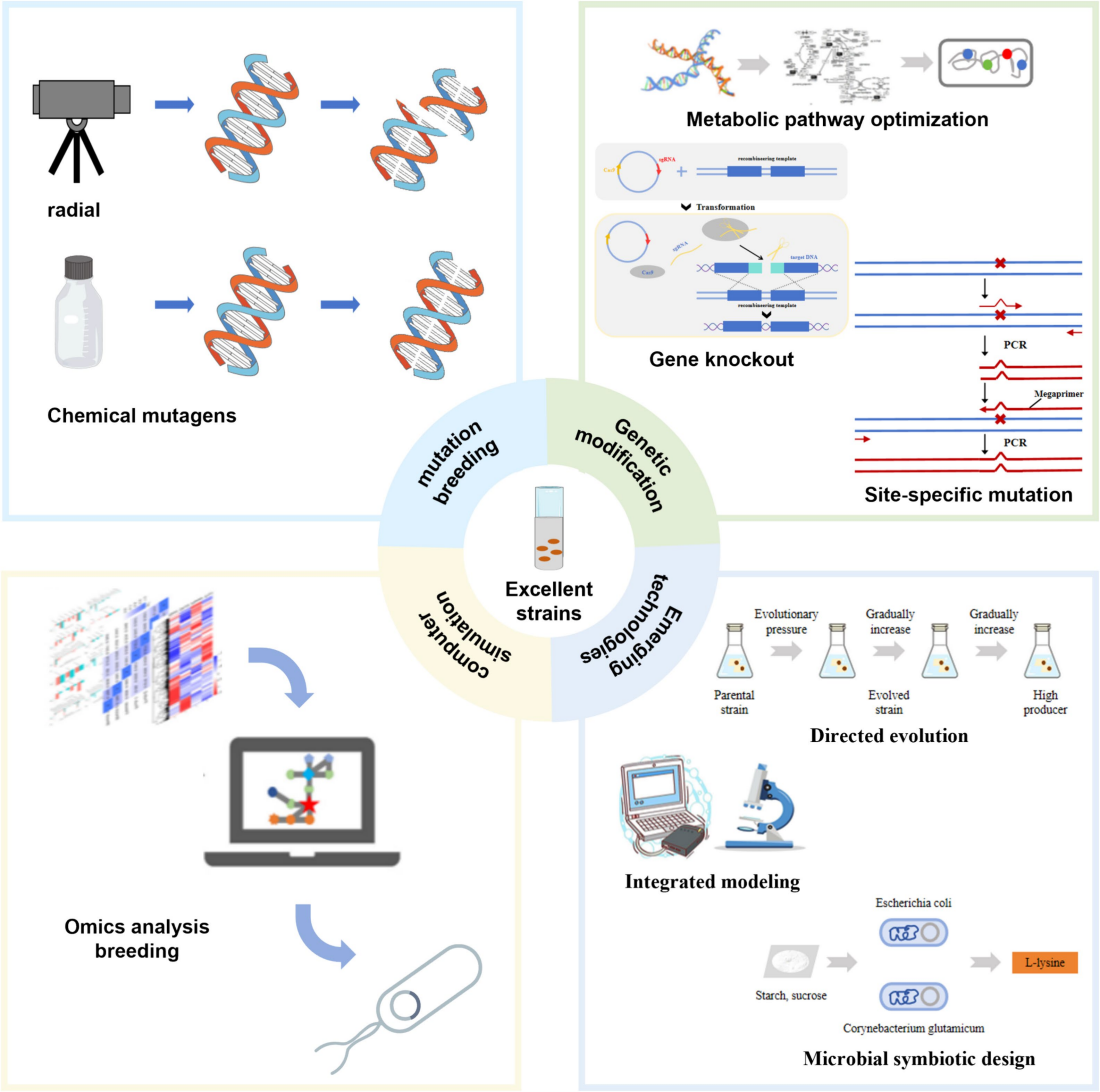
Strain transformation strategy. This figure illustrates various techniques for modifying *Escherichia coli* strains to increase L-lysine production.

Significant progress has been made in breeding *Escherichia coli* for lysine production using microbial consortia design. Researchers have combined genetically engineered lysine-assimilating *E. coli* with glutamate-assimilating *Corynebacterium glutamicum* to form a synthetic symbiotic consortium. In this alliance, *E. coli* secretes fructose as a carbon source, which *C. glutamicum* utilizes for growth and metabolism. Meanwhile, *C. glutamicum* synthesizes and secretes L-lysine, which is then utilized by *E. coli*. This mutualistic relationship effectively converts sucrose into L-lysine (Jia et al., 2016). This approach not only increases L-lysine production and reduces costs but also provides environmental sustainability benefits. By leveraging the metabolic capabilities of each organism, the consortium optimizes resource utilization and enhances overall process efficiency. Future research may further refine these microbial communities to expand their applications in biotechnology and industrial processes (Figure 3).

## 3 Optimization of fermentation process

Constructing or breeding superior L-lysine-producing strains is only the initial stage of industrial production. To fully realize the production potential of these strains, it is necessary to optimize the fermentation process to achieve higher production efficiency and product concentration.

### 3.1 Cultivation methods

The fermentation methods for L-lysine production by *Escherichia coli* mainly include continuous fermentation and fed-batch fermentation. In continuous fermentation, the fermentation broth is continuously replenished and removed, maintaining microbial growth in a stable state, thereby extending the production cycle and increasing L-lysine accumulation. This method is suitable for long-term efficient production but requires a high level of system stability, necessitating precise control of fermentation conditions. Researchers have developed intelligent control systems that enable automated process control by real-time monitoring of fermentation parameters such as pH, dissolved oxygen, and glucose concentration, further enhancing production stability and L-lysine yield. Additionally, improvements in the design of novel bioreactors, such as the application of membrane bioreactors, have significantly increased the efficiency of continuous fermentation (Chen et al., 2024b).

Fed-batch fermentation, on the other hand, involves the gradual addition of nutrients during the fermentation process to avoid substrate inhibition effects and the accumulation of by-products. Due to its flexibility and ease of control, fed-batch fermentation is widely used in industrial production. By finely tuning feeding strategies, fed-batch fermentation can significantly improve L-lysine yield and production efficiency (Rajpurohit and Eiteman, 2022). Researchers have explored various feeding strategies, such as tricalcium phosphate, sulfuric acid, and activated carbon pretreatment methods (TPSA), to enhance sugar consumption rates and L-lysine production. Moreover, multi-mode feeding strategies, such as staged feeding and multi-substrate feeding, have been developed to maximize yield and production efficiency (He et al., 2015).

### 3.2 Fermentation conditions

#### 3.2.1 Fermentation temperature

Fermentation temperature is one of the critical factors affecting lysine production efficiency in *Escherichia coli*. Temperature directly influences the metabolic activity, enzyme functionality, and growth rate of the cells. During lysine fermentation, a temperature of approximately 37°C is typically chosen, as it is the optimal growth temperature for *E. coli*, maximizing biomass accumulation and lysine yield. However, excessively high temperatures can intensify cellular stress responses and reduce enzyme activity, leading to a decrease in lysine production. Conversely, lower temperatures may slow metabolic rates but can help reduce by-product formation and extend the fermentation period. Therefore, precise temperature control is crucial for optimizing lysine production (Ma et al., 2021).

Recent advancements have shown that dynamic regulation of fermentation temperature can further enhance lysine production efficiency. For instance, maintaining a higher temperature during the initial phase of fermentation can promote rapid *E. coli* growth and early product accumulation, while lowering the temperature in the later stages can reduce by-product formation and prolong cell viability, thereby improving the final lysine yield. Additionally, some studies have explored the application of temperature-sensitive regulatory elements, enabling *E. coli* to automatically adjust its metabolic pathways in response to temperature changes, maximizing lysine synthesis. By utilizing computer simulations and systems biology approaches, researchers can predict cellular metabolic behavior under various temperature conditions, optimizing the fermentation process. The application of these dynamic temperature control strategies and intelligent regulatory methods holds significant promise for advancing the industrial-scale production of lysine.

#### 3.2.2 Dissolved oxygen in fermentation

Dissolved oxygen (DO) plays a crucial role in the fermentation process of L-lysine production by *Escherichia coli*, influencing cell growth, metabolic activities, and the final yield of L-lysine. *E. coli* is an aerobic microorganism, and during fermentation, an adequate oxygen supply can promote efficient carbon source metabolism, thereby increasing cell growth rates and product accumulation. However, excessively high DO levels may induce oxidative stress, inhibiting L-lysine synthesis, while insufficient DO can lead to the accumulation of metabolic byproducts and reduced fermentation efficiency. Therefore, maintaining an optimal DO level is essential for the efficient production of L-lysine by *E. coli*.

The intelligent monitoring and dynamic control of DO during fermentation have become focal points of research. Advanced sensor technology is utilized to monitor DO levels in real time, and in combination with computer control systems, enables dynamic adjustment of DO to meet the changing oxygen demands at different stages of the fermentation process (Zhou et al., 2011). Additionally, research has explored methods such as optimizing agitation speed, aeration rates, and employing microbubble oxygenation techniques to improve oxygen transfer efficiency, ensuring that *E. coli* obtains sufficient oxygen supply under high-density culture conditions.

#### 3.2.3 Fermentation pH

pH directly influences the physiological state of cells, enzyme activity, and the balance of metabolic pathways. During the initial stages of fermentation, a lower pH favors rapid growth of *Escherichia coli*; however, as fermentation progresses, the accumulation of L-lysine can lead to acidification of the culture medium. If not controlled, the resulting pH drop may inhibit cellular activity and reduce L-lysine yield. Therefore, maintaining an optimal pH is crucial for achieving efficient L-lysine production. Buffer systems can be employed to stabilize the pH during fermentation and minimize its impact on L-lysine production. For example, adding pH buffers like phosphate or adjusting the initial pH of the medium can provide high buffering capacity and consistent pH levels (Ferreira et al., 2015). The addition of weak bases such as calcium carbonate to neutralize fermentation acids can also help regulate pH. Additionally, the use of ammonia for neutralization is also another popular approach.

#### 3.2.4 Culture medium formula

The composition of the fermentation medium is crucial for enhancing lysine production in *Escherichia coli*. Traditional fermentation media typically consist of carbon sources, nitrogen sources, inorganic salts, trace elements, and vitamins. Common carbon sources include glucose, sucrose, and corn steep liquor, where the choice and concentration of the carbon source directly affect the growth rate of the microbial cells and lysine accumulation. Nitrogen sources usually involve yeast extract, soybean meal hydrolysate, or inorganic nitrogen (such as ammonium salts) to provide essential amino acids and other nitrogenous compounds necessary for cell growth. Additionally, inorganic salts and trace elements like phosphates, sulfates, calcium, magnesium, and zinc play vital roles in metabolic activities, while vitamins such as biotin and niacin are important for maintaining enzyme activity and promoting metabolism (Wang et al., 2023). Through optimization methods like response surface analysis, the optimal conditions for the medium composition can be systematically determined. In recent years, attention has also been given to non-traditional carbon sources, such as glycerol and cellulosic hydrolysates, as potential medium alternatives. These novel carbon sources not only enhance lysine yield but also offer advantages like reducing production costs and minimizing waste discharge. Furthermore, the development of *E. coli* specific media that can adapt to high-density fermentation, as well as multifunctional media formulations that integrate pH adjustment and foam control, has become a current research focus.

### 3.3 Extraction and purification of lysine

A critical factor in production is the separation and purification of lysine. Developing appropriate separation and extraction methods is essential for enhancing product yield and reducing production costs.

#### 3.3.1 Traditional separation methods

##### 3.3.1.1 Extraction methods: reverse micelle extraction and solvent extraction

Reverse micelle extraction and solvent extraction are among the most commonly used methods. Reverse micelle extraction is straightforward, allows for continuous operation, and produces products with high catalytic activity. However, its main limitation is that effective separation requires a very low ion concentration in the solution (Chaurasiya and Hebbar, 2017). Additionally, electrostatic interactions between surfactants and amino acid ions can affect extraction efficiency. In recent years, researchers have made progress in improving the stability and selectivity of reverse micelle systems. For instance, by developing novel gemini surfactants and multifunctional additives, it is possible to enhance the stability of reverse micelles and increase the extraction efficiency of lysine. Moreover, integrating reverse micelle extraction with membrane separation techniques is being explored to improve extraction efficiency and reduce energy consumption.

Solvent extraction leverages the solubility differences of lysine in various solvents, using organic solvents to extract lysine from the fermentation broth. Although solvent extraction is simple and requires relatively basic equipment, organic solvents pose health risks due to their volatility, and the separation efficiency may not be optimal (Sánchez-Velázquez et al., 2023). Recently, the development of environmentally friendly solvents has become a research hotspot in solvent extraction. The use of green solvents, such as ionic liquids and deep eutectic solvents, can reduce environmental pollution and improve lysine extraction efficiency. Additionally, researchers are exploring the combination of solvent extraction with other separation techniques, such as ultrasound-assisted extraction or microwave-assisted extraction, to further enhance extraction efficiency and yield.

##### 3.3.1.2 Acid precipitation

Acid precipitation involves adding acids (such as hydrochloric acid, sulfuric acid, or other organic acids) to the fermentation broth, lowering the pH, and causing lysine to precipitate out of the solution. The isoelectric point of lysine is typically around pH 9.5, and in an acidic environment, lysine molecules become positively charged, forming lysine salts with reduced solubility, leading to precipitation. Acid precipitation is simple to operate and effectively separates lysine in a short time. By studying the effects of different acids (organic and inorganic) and their concentrations on lysine precipitation, researchers have found that selective acid use and pH optimization can improve lysine precipitation efficiency and purity. For example, some studies suggest that using milder organic acids, such as citric acid, can reduce co-precipitation of impurities and increase lysine recovery. Combining acid precipitation with membrane separation can further purify lysine precipitates through membrane retention, thereby enhancing the purity of the final product (Chen et al., 2020). The use of more environmentally friendly acids or acidic solution systems can reduce environmental impact. Additionally, some studies have explored the possibility of recycling and reusing waste acids during the precipitation process to lower production costs and reduce waste liquid discharge. Real-time optimization of lysine precipitation processes can be achieved through online monitoring and feedback control of pH and precipitation conditions, ensuring consistent product quality.

##### 3.3.1.3 Ion exchange resin

Ion exchange resins are porous polymers with functional ionic groups that can separate charged lysine molecules from solutions through reversible ion exchange reactions. Cation exchange resins are typically used in lysine extraction because lysine behaves as a positively charged cation under most fermentation broth pH conditions. Ion exchange resins have high selectivity for lysine and operate under mild conditions. In recent years, researchers have developed resins with larger specific surface areas and higher ion exchange capacities, significantly improving lysine adsorption efficiency. Additionally, resins with special functional groups have been introduced to enhance lysine selective adsorption, reducing co-adsorption of impurities (Chen X. et al., 2019). The application of immobilized resins and multifunctional resins is becoming more common to improve overall process efficiency and stability. Recent studies have explored efficient resin regeneration methods, such as optimizing the composition of regeneration solutions and using auxiliary regeneration techniques (e.g., ultrasound or microwave assistance), to extend the lifespan of resins while maintaining their high efficiency (Zhang et al., 2011; Ecker et al., 2012; Li et al., 2012; Yokoyama et al., 2013).

##### 3.3.1.4 Membrane separation

Membrane separation technology uses membranes with specific pore sizes to separate different components in a solution. Depending on pore size and membrane characteristics, membrane separation technology is primarily divided into microfiltration (MF), ultrafiltration (UF), nanofiltration (NF), and reverse osmosis (RO) (Wang K. et al., 2022). Ultrafiltration and nanofiltration are commonly used in lysine extraction. Membrane fouling is a major bottleneck limiting the development of membrane separation technology. Recently, various anti-fouling membrane technologies have been developed, such as surface coating technology, dynamic membrane technology, and surface functionalization technology. These methods involve introducing hydrophobic, antibacterial, or superwetting coatings on the membrane surface, significantly reducing membrane fouling and improving the stability and efficiency of the separation process. Membrane bioreactors, which combine membrane separation technology with bioreactors, enable integrated operation during fermentation and separation extraction (Du Y. et al., 2022).

##### 3.3.1.5 Electrodialysis

Electrodialysis uses membranes with selective ion exchange functions (cation and anion membranes) to separate charged molecules like lysine from fermentation broth under the influence of a direct current electric field (Doble, 2016). Lysine exists as a positively charged ion in solution and moves through cation exchange membranes under an electric field, while other impurities pass through anion exchange membranes or are retained, thereby concentrating and purifying lysine. Electrodialysis is energy-efficient but requires precise control of operating conditions, such as current density and flow rate. Additionally, electrodialysis membranes are susceptible to fouling and scaling, which can reduce separation efficiency.

#### 3.3.2 Emerging separation technology

##### 3.3.2.1 Ultrasonic-assisted extraction

Ultrasonic-assisted extraction technology utilizes the cavitation effect generated by ultrasound waves propagating in liquid. This effect creates localized high temperatures and pressures, which disrupt the cell wall and increase the permeability of intracellular substances, thereby improving extraction efficiency (Shen et al., 2023). This method, known for its efficiency, energy-saving, and environmental-friendly characteristics, shows great potential in the extraction and separation of lysine produced by *Escherichia coli*.

##### 3.3.2.2 Enzymatic hydrolysis

Enzymatic hydrolysis refers to the selective degradation of the cell wall or membrane by enzymes, allowing the release of intracellular lysine for extraction and separation (Yi et al., 2023). This method offers high specificity and mild reaction conditions, avoiding the degradation of lysine or the formation of by-products that can occur during chemical extraction.

##### 3.3.2.3 Reverse-phase high-performance liquid chromatography

**Figure 4:**
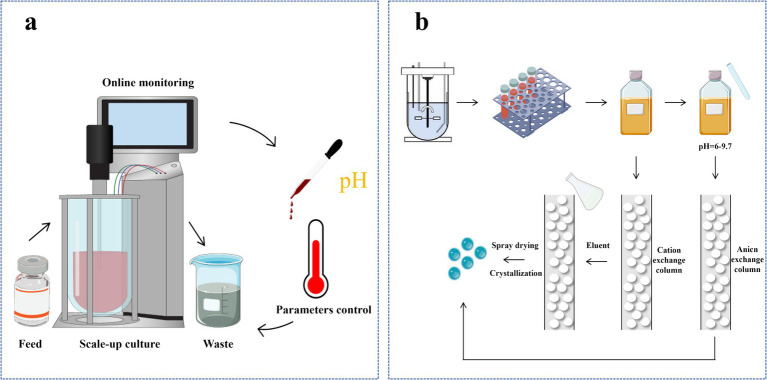
Production of L-lysine. **(a)** Continuous process of fermentation for L-lysine production; **(b)** Isolation and refinement of L-lysine fermentation broth.

Reverse-phase high-performance liquid chromatography (RP-HPLC) is a widely used method for the separation, purification, and analysis of biomolecules, including lysine. RP-HPLC separates lysine based on differences in the partition coefficients between the non-polar stationary phase and the polar mobile phase (Zhang et al., 2024). This method is renowned for its high resolution, excellent selectivity, and efficiency, enabling precise separation and quantitative analysis of lysine (Figure 4).

## 4 Lysine derivatives

Lysine is widely used as a nutritional supplement and chemical intermediate in the beverage, feed, and food industries. Additionally, lysine holds promise as a precursor for the production of high-value chemicals used in pharmaceuticals, active pharmaceutical ingredients, and materials.

### 4.1 Lysine derivatives for bio-based materials

Putrescine (1,5-diaminopentane) has multiple uses in both industrial and agricultural fields. Bio-based putrescine is an important platform chemical for bio-based polyamides such as PA 54, PA 510, and PA 512 (Kim et al., 2017). The production of putrescine can be achieved through whole-cell biocatalysis or microbial fermentation, involving recombinant *Escherichia coli* and *Corynebacterium glutamicum* (Gevrekci, 2017). Engineered strains have demonstrated increased putrescine yield, with *E. coli* achieving a maximum yield of 9.61 g/L (Li et al., 2020). Additionally, whole-cell biotransformation is a feasible method for efficiently synthesizing putrescine from L-lysine.

5-Aminovaleric acid (5AVA) is a precursor for the production of nylon 5, nylon 6,5, and other five-carbon derivatives such as valerolactam, glutaric acid, 1,5-pentanediol, and 5-hydroxyvaleric acid. It is a promising platform compound (Liu et al., 2014). These compounds have widespread applications in plastics, solar cells, and artificial antigens. Various biosynthetic pathways have been explored for the production of 5AVA. One approach involves the natural pathway of *Pseudomonas putida*; another method uses a biosynthesis route mediated by *δ*-aminovaleramidase (DavA) and Lys 2-monooxygenase (DavB), starting from L-lysine, which is first converted to 5-aminovaleramide and then hydrolyzed to 5AVA. This strategy has been implemented in engineered *E. coli*, achieving a final yield of up to 57.52 g/L (Jorge et al., 2017). Additionally, a novel pathway involves using Trichoderma reesei L-lysine *α*-oxidase (LysOx) to oxidize the α-carbon of L-lysine esters to 6-amino-2-ketohexanoic acid, which is then decarboxylated to produce 5AVA without the need for catalase.

δ-Valerolactam is an organic compound typically obtained by cyclization and dehydration of 5-aminovaleric acid (5AVA) under vacuum conditions (Park et al., 2014). These compounds are used as precursors for nylon 5 and nylon 6,5. Recombinant *E. coli* strains overexpressing Streptomyces acyl-CoA ligase ORF26 produce δ-valerolactam and *ε*-caprolactam (Zhang et al., 2017). Additionally, an efficient metabolic pathway for synthesizing ε-caprolactam and δ-valerolactam from *ω*-amino acids was constructed by activating *β*-alanine CoA transferase (Act) from *Clostridium propionicum* (Chae et al., 2017). Another innovative L-PA-based approach involves converting L-PA to δ-valerolactam under the catalysis of oxidative decarboxylase.

ε-Caprolactam is the starting material for the synthesis of nylon 6,5 and is also a primary chemical used in the synthesis of nylon 6. Researchers have developed a route for the chemical conversion of biomass-derived lysine into ε-caprolactam (Kumar et al., 2019). This process involves cyclization and subsequent transformation steps to produce α-amino-ε-caprolactam, which can be further utilized in the preparation of nylon 6. Additionally, ε-caprolactam can be produced by cyclization of purified biosynthetic intermediates, a method that holds promise for replacing the conventional process of producing ε-caprolactam from petroleum.

### 4.2 Lysine-based compounds for pharmaceuticals

L-Pipecolic acid (L-PA) is a crucial non-proteinogenic amino acid widely used as a key intermediate in the synthesis of various pharmaceuticals and biochemical compounds. Currently, L-PA synthesis is primarily achieved through two methods: biosynthesis and chemical synthesis. Due to the cumbersome procedures and low yields associated with chemical synthesis, biosynthesis has garnered significant attention from global chemical companies for its efficiency and environmental benefits. There are four known biosynthetic pathways for L-PA, including the production of L-PA through α-nitrogen loss and ε-nitrogen condensation from lysine, as well as through ε-nitrogen loss with the incorporation of lysine and α-nitrogen. These methods offer economically viable routes for the large-scale production of chiral L-PA.

6-Aminocaproic acid (6ACA) is a widely used non-natural amino acid known for its ability to inhibit the activity of enzymes such as plasmin, pepsin, and elastase, thereby effectively treating certain hemorrhagic conditions (Grottke et al., 2013). Additionally, 6ACA is a crucial component in the production of Nylon 6. Producing 6ACA from biological sources, particularly from lysine, has the potential to significantly reduce refinery emissions and enhance biosecurity. Although various biosynthetic pathways have been explored, further experimental validation of these engineered approaches is necessary.

## 5 Conclusion

In this review, we have explored various strategies for optimizing *Escherichia coli* strains and fermentation processes to enhance L-lysine production. Key approaches include genetic engineering of metabolic pathways, modification of regulatory mechanisms, and optimization of fermentation conditions. By targeting bottlenecks in the L-lysine biosynthetic pathway, significant improvements in yield and productivity have been achieved. Additionally, advances in synthetic biology and genome editing technologies, such as CRISPR/Cas9, have opened new avenues for more precise and efficient strain engineering (Pu et al., 2023).

Despite these advancements, there remain several challenges that need to be addressed. One major hurdle is the balance between growth and production, as maximizing L-lysine output often compromises the overall fitness of the microbial cells (Liu and Wang, 2021). Moreover, the complex interplay between metabolic fluxes and regulatory networks in engineered strains can lead to unexpected outcomes, requiring further fine-tuning and iterative testing (Pang et al., 2022). In large-scale industrial production, rising production costs, increasing environmental pollution, and high substrate consumption pose significant challenges. Current separation and purification processes require substantial amounts of acid–base reagents and water, leading to escalating costs and environmental burdens, which result in resource waste and hinder the development of lysine production. Membrane separation technology, with its advantages of energy savings, no phase transition, simplicity of use, scalability, and elimination of chemical reagents, can significantly enhance the separation and purification of L-lysine, thereby facilitating green production for enterprises (Zhang et al., 2019).

Looking forward, future research should focus on integrating systems biology approaches with machine learning and artificial intelligence to predict and design more efficient production strains. Additionally, exploring co-culture systems and dynamic control strategies could provide new opportunities for overcoming limitations associated with single-strain fermentations (Van Lent et al., 2023). With the continuous advancement of metabolic engineering technologies, we can anticipate more innovative approaches to further improve the yield and quality of L-lysine.
